# First record of *Gyrosteus mirabilis* (Actinopterygii, Chondrosteidae) from the Toarcian (Lower Jurassic) of the Baltic region

**DOI:** 10.7717/peerj.8400

**Published:** 2020-01-22

**Authors:** Jahn J. Hornung, Sven Sachs

**Affiliations:** 1 Niedersächsisches Landesmuseum Hannover, Hannover, Germany; 2 Naturkunde-Museum Bielefeld, Abteilung Geowissenschaften, Bielefeld, Germany

**Keywords:** Chondrosteidae, *Gyrosteus mirabilis*, Toarcian, Lower Jurassic, Northern Germany

## Abstract

An isolated hyomandibula from a lower Toarcian carbonate concretion of the Ahrensburg erratics assemblage (Schleswig-Holstein, northern Germany) represents the first record of a chondrosteid fish from the Lower Jurassic of the southwestern Baltic realm. Except for its smaller size, the specimen is morphologically indistinguishable from corresponding elements of *Gyrosteus mirabilis* from the Toarcian of Yorkshire, England. This find, which probably originates from the western Baltic basin between Bornholm Island (Denmark) and northeastern Germany, markedly expands the known range of this chondrosteid taxon across the northern part of the strait connecting the Boreal Sea with the Tethys Ocean during the Early Jurassic. For the first time the extension of the paleogeographic range of a chondrosteid species beyond its type area is documented, which can contribute to future studies of vertebrate faunal provincialism during the Lower Jurassic in Europe.

## Introduction

The Ahrensburg erratics assemblage, named after its main occurrence in Pleistocene glacigenic deposits near the town of Ahrensburg, Schleswig-Holstein, northern Germany, provides a high diversity of mainly Lower Jurassic invertebrate and vertebrate fossils, often in exceptional quality (Lehmann, 1967, 1971; Lierl, 1990; Sachs et al., 2016). They open a precious window into the marine fossil communities of the southwestern Baltic and adjacent regions, in which autochthonous surface exposures of Lower Jurassic strata are rare and mostly covered by Quarternary deposits. The specimens are preserved in calcareous concretions formed during early diagenesis in a clayey or marley sedimentary environment, that intercalates with fine-grained sandstones. The formation of the concretion often provided an uncompressed, three-dimensional preservation and the record of delicate details (Lehmann, 1967). Aside of rare remains of aquatic and terrestrial tetrapods (Von Huene, 1966; Lehmann, 1971; Sachs et al., 2016), the Ahrensburg assemblage also yielded a marine fish fauna typical for the Lower Jurassic, which has been partly figured by Lehmann (1971) and Lierl (1990). The composing Lower Jurassic marine sediments show close relationships to coeval deposits from NE-Germany (Toarcian of Dobbertin and Grimmen, Mecklenburg-Vorpommern). The fish fauna of these outcrops, as currently known, is summarized in Table 1.

**Table 1:** Synoposis of the Toarcian fish fauna from the Lower Toarcian of northern Germany.

Taxon	Locality			References
	Ahrensburg	Dobbertin	Grimmen	
**ELASMOBRANCHII**				
**Hybodontiformes**				
Hybodontidae gen. et sp. indet.	+		+	Lehmann (1971) and Ansorge (2007)
**ACTINOPTERYGII**				
**Acipenseriformes**				
*Gyrosteus mirabilis* Woodward (1889)	+			This work
**Saurichthyformes**				
*Saurorhynchus hauffi* Maxwell & Stumpf (2017)			+	Maxwell & Stumpf (2017)
**Lepisosteiformes**				
*Lepidotes* spp.^1^	+	+	+	Jaekel (1929a), Malzahn (1963), Lehmann (1971), Thies (1989), Obst et al. (2015), and Thies, Stevens & Stumpf (2019)
**Dapediiformes**				
*Dapedium* cf. *punctatum* (Agassiz, 1835)		+		Obst et al. (2015)
*Tetragonolepis* cf. *semicinctus* Bronn (1830)			+	Zessin & Krempien (2010)
**Pycnodontiformes**				
*Grimmenodon aureum* Stumpf et al. (2017)			+	Stumpf et al. (2017)
**“Pholidophoriformes”**				
*Grimmenichthys ansorgei* Konwert & Hörnig (2018)			+	Konwert & Hörnig (2018)
**Pholidophoriformes**				
*Pholidophorus* sp.	+			Lehmann (1971)
Pholidophoridae gen. et sp. indet.			+	Konwert & Hörnig (2018)
**Leptolepiformes**				
*Leptolepis coryphaenoides* (Bronn, 1830)		+	+	Konwert & Stumpf (2017)
*Leptolepis normandica* Nybelin (1962)		+		Konwert & Stumpf (2017)
*Leptolepis jaegeri* Agassiz (1832)		+	+	Konwert & Stumpf (2017)
*Leptolepis* sp.	+^2^		+	Lehmann (1971) and Konwert & Stumpf (2017)

**Notes:**

1 There are probably more then one species of *Lepidotes* in the material, but the alphataxonomy has not yet been resolved sufficiently (Thies, Stevens & Stumpf, 2019).

2 Mostly disarticulated material referred to *L*. “*bronni”* (=*L. coryphaenoides*) by Lehmann (1971), without more detailed description.

Here we describe for the first time the occurrence of a chondrosteid fish from the Baltic region that adds to the known occurrence of this group in southern Germany and the UK.

### Geological context

**Figure 1:**
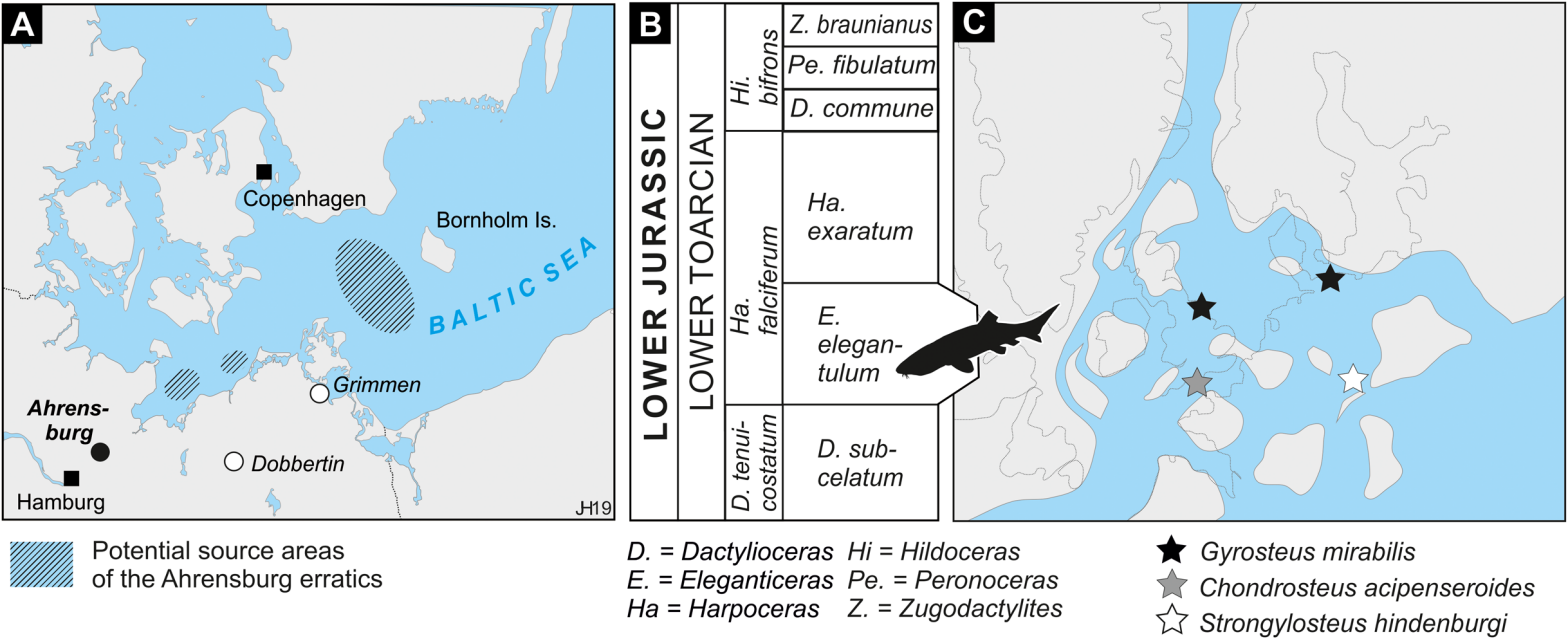
Location, stratigraphic, and paleogeographic information. (A) Location map. Potential source areas for the Ahrensburg erratics from Sachs et al. (2016). (B) Biostratigraphical framework of the Toarcian in the southwestern Baltic realm (based on Lehmann, 1968). Silhouette of chondrosteid fish is based on artwork © N. Tamura. (C) Occurrences of chondrosteids projected on the paleogeography of the North-West European Epicontinental Archipelago during the Toarcian. Present-day shorelines in dotted lines for orientation. Based on the plate tectonic reconstruction by Scotese (2013) and paleogeography by Cox & Sumbler (2002) and Xu et al. (2018).

The exact origin of the erratics from the Ahrensburg assemblage is yet unknown but probably located in the southwestern Baltic Sea. Similarities in lithofacies as well as the consideration of late Pleistocene ice-flow directions, indicate the region off the shore of Mecklenburg-Vorpommern (Fig. 1A) as the most plausible source area. The Ahrensburg erratics assemblage (“Ahrensburger Geschiebesippe,” Ernst, 1938) consists of a suite of sedimentary strata that range from the Lower Jurassic through the Lower Cretaceous (Berriasian), but is dominated by fossiliferous concretions of Toarcian age, that can be easily distinguished by petrographic characters and fossil content (Lierl, 1990). The Toarcian source rocks probably form part of a prodeltaic facies belt in the southwestern Baltic Basin, that also comprises the deposits of Dobbertin and Grimmen onshore in northeastern Germany. The latter have recently been included in the Ciechocinek Formation stretching eastward into Poland and represent a more marginal setting compared to the basinal deposits of the Whitby Mudstone Formation of Yorkshire and the Posidonienschiefer Formation of southern Germany (Mönnig, Franz & Schweigert, 2018). The proximal facies of this delta is partly exposed on Bornholm Island (Denmark, for details and discussion see Sachs et al. (2016) and references therein).

The fossiliferous Lower Jurassic concretions of the Ahrensburg assemblage contains ammonites ranging in age from the lowermost Toarcian *Dactylioceras tenuicostatum* zone to the upper Toarcian *Grammoceras thouarsense* zone (Lehmann, 1968; Lierl, 1990). However, the vertebrate fossils from Ahrensburg, Grimmen and Dobbertin concentrate in the *Harpoceras falciferum* zone of the lower Toarcian (Figs. 1B and 1C; Lierl, 1990; Ansorge, 2007; Obst et al., 2015; Sachs et al., 2016) with most of the fossiliferous deposits of the Ahrensburg assemblage dating to the *Eleganticeras elegantulum* subzone (Lehmann, 1968; Obst et al., 2015). The succession in Dobbertin spans the *elegantulum* subzone and possibly into the overlying *Harpoceras exaratum* subzone (Obst et al., 2015), while in Grimmen the main vertebrate occurrences are located in the *Ha. exaratum* subzone (Ansorge, 2007).

## Results

### Systematic paleontology

Actinopterygii Cope, 1887

Acipenseriformes Berg, 1940

Chondrosteidae Egerton, 1858

*Gyrosteus* Woodward, 1889 (ex Agassiz, 1834)

*Type species: Gyrosteus mirabilis* Woodward, 1889 (ex Agassiz, 1834)

***Gyrosteus mirabilis*Woodward, 1889 (ex Agassiz, 1834)**

**Figure 2:**
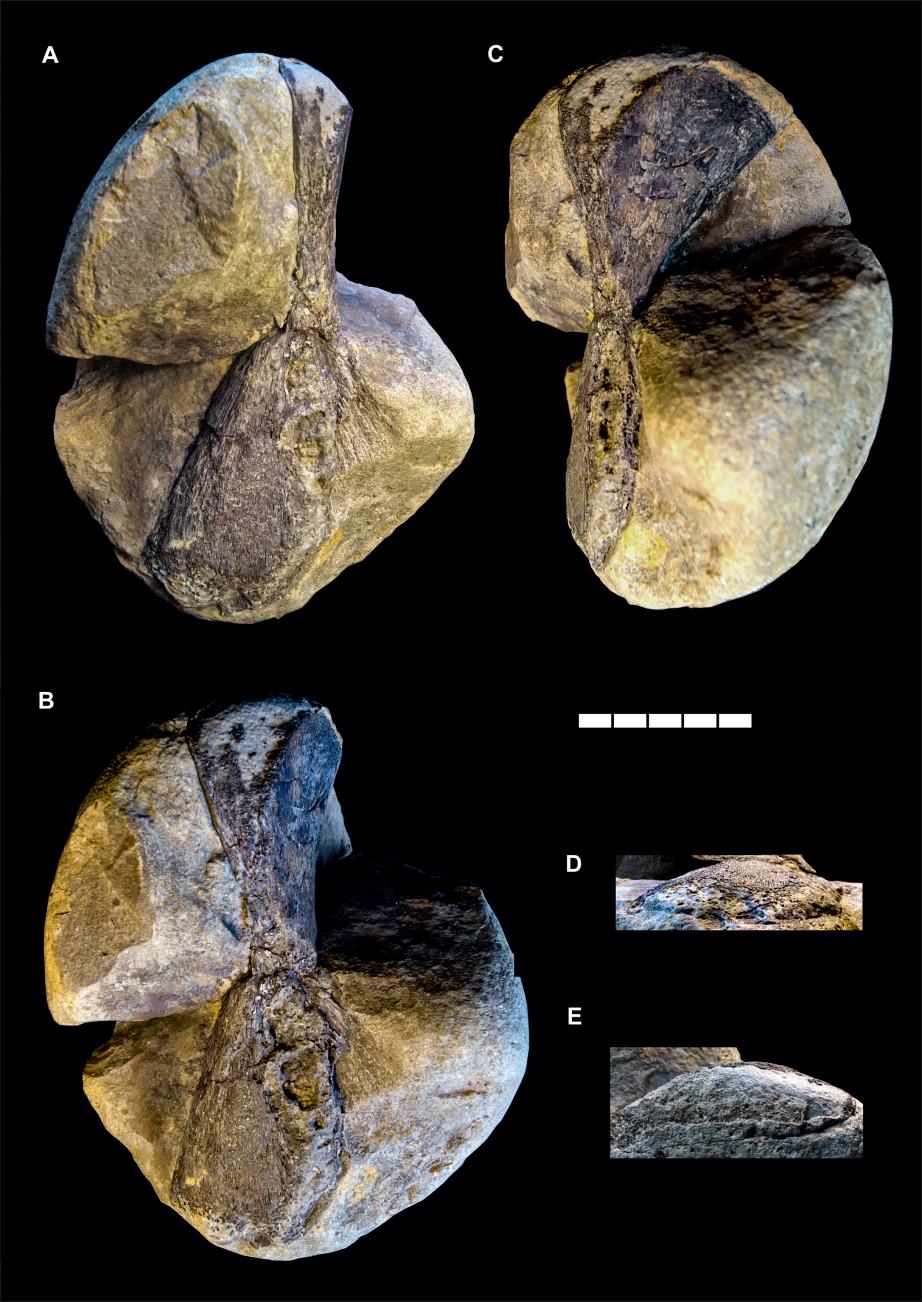
*Gyrosteus mirabilis* Woodward (1889). GPIH 4864, right hyomandibula. Lower Toarcian (*elegantulum* subzone) of the Ahrensburg erratics assemblage; Ahrensburg, Schleswig-Holstein, northern Germany. In (A) lateral, (B) anterolateral, (C) anterior, (D) distal and (E) proximal views. Illumination from the lower right in (A) and (C), from the lower left in (B), and from the upper right in (D) and (E). Scale bar represents five cm.

**Figure 3:**
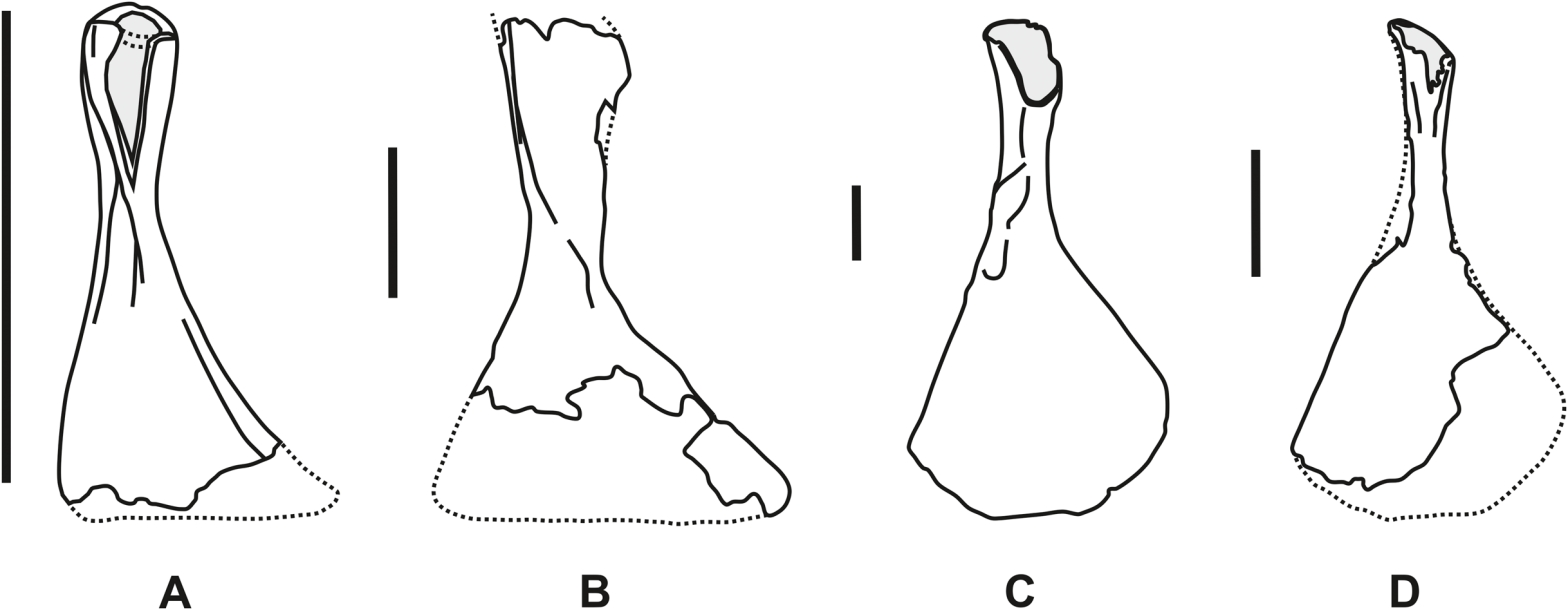
Comparative outline of the right hyomandibula in lateral view. (A) *Chondrosteus acipenseroides* (after Egerton, 1858). (B) *Strongylosteus hindenburgi* (after Hennig, 1925). (C) and (D) *Gyrosteus mirabilis*. (C) NHMUK P 3356a (after Woodward, 1890) and (D) GPIH 4864. Scale bar represents 50 mm.

*Material:* GPIH 4864: right hyomandibula (Figs. 2 and 3D; Table 2).

**Table 2:** Measurements (in mm) of GPIH 4846.

**Proximal epiphysis**	
Preserved max. medio-lateral width	72
Preserved max. antero-posterior width	18
**Mid-shaft section**	
Max. diameter	20
Min. diameter, perpendicular to the max. diameter	12
**Distal epiphysis**	
Preserved max. medio-lateral width	6
Preserved max. antero-posterior width	76

*Locality and horizon:* The specimen derives from an erratic of the Ahrensburg assemblage (“Ahrensburger Geschiebesippe,” Ernst, 1938), displaced during the Pleistocene to the area of Ahrensburg near Hamburg, Schleswig-Holstein, N-Germany. The petrographic characters of the grayish (weathering to yellowish), concretionary, sandy calcareous marlstone suggest an origin from the lower *falciferum* zone (*elegantulum* subzone), lower Toarcian, Lower Jurassic (Lierl, 1990).

## Description

The material (Fig. 2) consists of a single element preserved in a marlstone concretion and exposed only partially in posterior and lateral aspect. The overall shape of the bone is that of a flat hourglass with two expanded ends and a strongly constricted mid-shaft region. The dorsal and and ventral extremities as well as the posteroventral margin are damaged and incomplete. The dorsal and ventral epiphyses are twisted by about 70° relative to each other in the long axis of the bone. The ventral epiphysis is flat and fan-shaped, the anterior and posterior margins are straight in the preserved portions and diverge by an angle about 60° from each other. The anterior margin is broken off but its outline is partially preserved as an impression. The lateral surface is smooth, very gently concave and converges with the medial surface gently in distal direction. The medial surface shows a weak radial, dorsoventral sculpture in the area of the impression, otherwise it is concealed by matrix. The mid-shaft region is very short, the diverging margins of the dorsal epiphysis meet those of the ventral epiphysis immediately, without an straight-sided intersection. The cross-section of the mid-shaft is compressed, squared with rounded edges.

The proximal epiphysis expands gently anteroposterioly. The lateral margin is slightly eroded and diverges only very gently laterally. The medial margin diverges more pronounced, resulting in a concave medial profile and a distinct medial expansion of the head of the epiphysis. The dorsal surface is damaged but not as incomplete as in the ventral epiphysis. The anterior surface forms a rounded angle with the medial surface, both are smooth.

While the midshaft region exhibits the presence of a substantia spongiosa, other parts of the element, especially towards the extremities lack an internal osseous tissue and are filled with sediment. This indicates that the bone was not fully ossified and substantial parts of the epiphyses were cartilaginous.

Measurements are summarized in Table 2.

## Discussion

### Comparative discussion of GPIH 4864

The overall morphology and ossification pattern of GPIH 4864 allow its identification as hyomandibula of a large-bodied, chondrosteid fish. Unambiguous members of the family Chondrosteidae are known exclusively from the Lower Jurassic of Central and Western Europe (England and Germany, Grande & Bemis, 1991, 1996; Bemis, Findeis & Grande, 1997; Hilton & Forey, 2009). Currently three genera are known (Fig. 3), however, the validity of the genera *Gyrosteus* and *Strongylosteus* Jaekel (1929b) are disputed and some authors suggest their synonymization with *Chondrosteus* Egerton, 1858 (ex Agassiz, 1834) (compare Bemis, Findeis & Grande, 1997). Pending a revision of *Strongylosteus hindenburgi* and *G. mirabilis*, these genera are accepted valid for the purpose of this work. *Chondrosteus* and *Strongylosteus* are monospecific. For *Gyrosteus*, a referred species, *G. subdeltoideus* Stinton & Torrens, 1968, has been described, based upon an otolith from the Bathonian of Leicester, England. However, the otoliths of the type species, *G. mirabilis*, are unknown and the generic assignment by Stinton & Torrens (1968) was based solely on the similarity of the otolith to those of acipenseriforms and stratigraphic grounds. *“G.” subdeltoideus* can therefore not reliably be referred to *Gyrosteus*.

*Chondrosteus acipenseroides* Egerton, 1858 (ex Agassiz, 1834) (syn. *C. crassartus* Egerton, 1858, *C. pachyurus* Egerton, 1858; compare Hilton & Forey, 2009, see also Traquair, 1877, 1887; Davis, 1887; Fig. 3A)—Sinemurian Black Ven Mudstone Member, Charmouth Mudstone Formation, of Dorset (S-England, Forey, Longbottom & Mulley, 2010). Complete specimens with a standard length of up to one meter are known (Egerton, 1858; Hilton & Forey, 2009). Individuals of this taxon are therefore markedly smaller than those of the other chondrosteid species. In average the hyomandibula is only 3–5 cm long. Aside from the size, differences of the element to the specimen from Ahrensburg include the morphology of the dorsal process which is ovate in cross-section at its expanded end and shows only slight anteroposterior compression. The dorsal epiphysis is not ossified and the dorsal process is only slightly axially rotated relatively to the ventral epiphysis. The former also bears a lateral ridge, that is, not present in the following species.

*Strongylosteus hindenburgi* (Hennig, 1925) (Fig. 3B)—Lower Toarcian Posidonienschiefer-Formation of Baden-Württemberg (SW-Germany). The authorship of this species has been stated previously as “Pompeckj, 1914” (Grande & Bemis, 1991). In fact, “*Chondrosteus hindenburgi”* was first mentioned (as a nomen nudum) by Hauff (1921), announcing a description of this taxon in a forthcoming paper by Josef F. Pompeckj—a publication that apparently never went into print. Hennig (1925) noted that Pompeckj labeled the future holotype specimen on its acquisition by the Tübingen collection in 1914 as “*Chondrosteus hindenburgi*,” and regarded Hauff (1921) as the first appearance of the name in print. He suggested Pompeckj’s species name “to be retained” and cited him as the species’ author, but it was only his own description that formally established the species.

This taxon is known from complete specimens with a standard length up to three meters (Hennig, 1925). The size of the hyomandibula resembles the specimen from Ahrensburg more than *C. acipenseroides* but some morphological differences are present, namely at the dorsal epiphysis. In *S. hindenburgi* the shaft expands only gently towards its dorsal end, the maximum mediolateral width of the head amounts to *c*. 175% of the minimum shaft diameter. The twist in the long axis of the element is much smaller than 70°. The dorsal epiphysis is crushed in all known specimens due to incomplete ossification, rendering the reconstruction of its cross-section uncertain. It was probably ovate to elliptical. The ventral epiphysis is not fully exposed due to overlying skull elements. While in the Ahrensburg specimen the shaft is very short, it is proportionally longer and more slender with almost parallel sides in *S. hindenburgi*.

*Gyrosteus mirabilis* Woodward, 1889 (ex Agassiz, 1834) (Figs. 3C and 3D)—Lower Toarcian Whitby Mudstone Formation of Yorkshire (NE-England). The taxon was first created by Agassiz (1834) as a nomen nudum. It was mentioned but not described in subsequent publications by Egerton (1837) and Morris (1854). Blake (in Tate & Blake, 1876: 256, pl. II, Figs. 2 and 3) provided more details and figured the taxon for the first time, but only Woodward (1889) described it thoroughly and figured key elements in detail. Like *S. hindenburgi*, *G. mirabilis* were large to very large fishes. Woodward (1890) estimated a maximum standard length of six to seven meters and reported a hyomandibula with a length of about 50 cm. This taxon is known solely from isolated elements, including several hyomandibulae (Woodward, 1889, 1890, 1895, 1899). They exhibit extensive ossification and a strong anteroposterior compression of the dorsal epiphysis. In mediolateral direction the dorsal epiphysis is strongly expanded with a convexly rounded lateral, and an angular protruding dorsomedial corner. This results in a slightly asymmetric, paddle-shaped dorsal epiphysis. The latter is connected to the ventral epiphysis via a very short tin and strongly tapering shaft. Both epiphyses are rotated axially against each other along the shaft by 70°–80°.

These features correspond with the Ahrensburg hyomandibula, especially the asymmetrically flattened, transversely widened dorsal process and the strong tapering and rotation of the shaft axis. These similarities allow a referral of the Ahrensburg specimen to *G. mirabilis*, a view further supported by the noted differences to the other chondrosteids and the stratigraphic age of the specimen. The comparatively small size may indicate an immature individual, which is corroborated by the partially incomplete ossification.

### Implications for paleozoogeography

Maisch & Ansorge (2004) suggested a strong faunal provincialism for marine amniotes in the lower Toarcian of Central Europe. According to these authors paleogeographic barriers separated a “British faunal province” (represented by taxa of the Yorkshire Basin) from a “Germanic faunal province” (represented by taxa from southern Germany). Both provinces were considered to be connected by an intermediate “Subgermanic faunal province” in the area of eastern France and Benelux. Sachs et al. (2016), following an analysis of the marine reptiles from Ahrensburg, concluded that the Baltic region showed more faunal similarities to the “Germanic faunal province” then to the “British faunal province.” However, *G. mirabilis* obiviously contributes an element of the “British faunal province” to the Baltic region, while *S. hindenburgi* from southern Germany can be considered as a representative of the “Germanic faunal province.” Possible explanations for this pattern are that the fish fauna had a different provincialism than the amniote communities, or that the Baltic region may represent an interdigitating zone between the “British” and “Germanic provinces.” A third alternative would be that the supposed vertebrate provincialism is an overinterpretation based on a too small sample of available material. Currently our knowledge about regionalism in the Lower Jurassic fish fauna is too minor to come to a decisive conclusion, and more finds and research are necessary.

## Conclusion

The rare and only incompletely known *G. mirabilis* is here described for the first time from the Toarcian of the southwestern Baltic realm. It originates from a prodeltaic facies, most probably deposited between the Danish island of Bornholm and the German Baltic coast (Lierl, 1990; Sachs et al., 2016). The relatively small size of the hyomandibula compared to the largest English material (ca. 40% smaller) may be related to a younger ontogenetic stage of the individual. Unfortunately, the specimen does not provide any new morphological information. However, it documents for the first time the extension of the paleogeographic range of a chondrosteid species beyond its type area. The addition of this interesting element to the Toarcian fish fauna of the marine strait between the Boreal Sea and the Tethys Ocean is an important contribution for future studies of vertebrate faunal provincialism during the Lower Jurassic in Europe.

## Institutional abbreviations

GPIH: Geomatikum, Geologisch-Paläontologisches Institut der Universität Hamburg, Hamburg, Germany
NHMUK: Natural History Museum, London, United Kingdom

## Additional Information and Declarations

### Data Availability

The following information was supplied regarding data availability: GPIH 4864 is stored in the Geomatikum, Geologisch-Paläontologisches Institut der Universität Hamburg, Hamburg, Germany.
